# Feasibility of a “No-Biopsy” Approach for the Diagnosis of Celiac Disease in Symptomatic Adults

**DOI:** 10.7759/cureus.54578

**Published:** 2024-02-20

**Authors:** Parul Punia, Kiran Bala, Mansi Verma, Ankita Nandi, Parveen Mahlotra, Sunita Singh, Seema Garg, Aparna Parmar, Devender Kumar

**Affiliations:** 1 Microbiology, Pandit Bhagwat Dayal Sharma Post Graduate Institute of Medical Sciences, Rohtak, IND; 2 Gastroenterology, Pandit Bhagwat Dayal Sharma Post Graduate Institute of Medical Sciences, Rohtak, IND; 3 Pathology, Pandit Bhagwat Dayal Sharma Post Graduate Institute of Medical Sciences, Rohtak, IND; 4 Microbiology, BPS Government Medical College for Women, Sonepat, IND; 5 Oral Medicine, Pandit Bhagwat Dayal Sharma Post Graduate Institute of Medical Sciences, Rohtak, IND

**Keywords:** anti-ttg antibodies, adults teenagers, marsh grading, biopsy, celiac disease

## Abstract

Celiac disease (CD) is an immune-mediated enteropathy, caused by hypersensitivity to gluten in genetically predisposed individuals. The worldwide prevalence of CD has been estimated to be approximately 1%. Most guidelines for diagnosis of CD rely on a sequential approach, with serological testing of antibodies against tissue transglutaminase (tTG) as a first-line test, followed by a duodenal biopsy. However, GI biopsy is an invasive procedure with various complications. Hence, this study was planned to ascertain whether it could be possible to have a non-biopsy approach, using only serological markers to establish the diagnosis of CD in adults.

Material and methods: It was a retrospective analysis of medical records of all biopsy-diagnosed CD patients with available anti-tTGA antibodies reports from 2019 to 2023. The patients were divided into three groups based on Marsh grading and anti-tTGA antibody levels were compared using various statistical tests.

Results: A total of 94 biopsy-diagnosed symptomatic CD patients with anti-tTGA antibody reports available formed the study group. Of these, 54 had biopsy findings consistent with Marsh 3 lesion, three had Marsh 2 lesion, and 37 had Marsh 1 lesion. A significant correlation existed between Marsh grading 3 lesion and anti-tTGA antibody levels above the upper limit of normal (ULN) x 10.

Conclusion: Serum levels of anti-tTGA antibodies greater than 10 x ULN can be used to identify symptomatic patients with Marsh grade 3 CD lesions.

## Introduction

Celiac disease (CD) is a chronic systemic autoimmune disorder where the immune system is sensitive to dietary gluten and related prolamines, resulting in small intestinal mucosal inflammation as well as other systemic extraintestinal manifestations in genetically susceptible individuals [1]. The global prevalence of CD varies in different geographic locations ranging between 0.5% and 1% in Europe and North America, approximately 1% in India, 2%-3% in Sweden and England, and even higher in Arab countries [2,3]. Despite being a major global public health issue, it is noteworthy that only 25% of affected individuals get diagnosed, putting the majority of missed CD patients at risk for various complications associated with the disease. In many developing countries including India, not more than 5% of individuals with CD get diagnosed and treated which is worrisome [4,5].

Currently, the diagnosis of CD in symptomatic individuals relies on a sequential approach, comprising serology including testing for antibodies against endomyseal antigen/tissue transglutaminase (EMA/tTG), followed by small bowel biopsy (SBB) as the confirmatory diagnostic test. Further, HLA DQ2/DQ8 testing is done in case of any discordance observed in the results. All major gastrointestinal organizations tend to consider the combined use of biopsy findings and positive serology as mandatory before initiating a gluten-free diet (GFD) for CD patients [6]. But ESPHAGN guidelines published in 2012 were the first ones to allow the diagnosis of CD without the need for intestinal biopsy in the pediatric population [7]. As per these guidelines, a “no biopsy” approach may be followed in symptomatic pediatric populations fulfilling the following criteria: high levels of TGA antibodies above 10 times the upper limit of normal (ULN), a positive EMA test in a second blood sample and presence of specific HLA DQ2 or DQ8 genes. Later on, the requirement for HLA testing was also removed from the guidelines [7].

The development of extremely sensitive and equally specific serological tests for screening of CD in the last two decades has evoked many researchers to evaluate their significance and feasibility in diagnosing CD without the need for a biopsy. Moreover, there are certain concerns regarding biopsy/histological diagnosis of CD in clinical practice including the following.

Poorly tolerated by patients, expensive, and carries risks (GI hemorrhage and perforation) [8]. Interobserver disagreements in the interpretation of SBB amongst pathologists [9]. Histologic findings are characteristic but not specific for CD [10]. Poor compliance for seropositive patients to undergo biopsy [11]. Unavailability of expertise at primary care centers especially in resource-limited settings.

Taking into concern these factors and considering the availability of extremely sensitive and specific serological tests, many studies have evaluated different cut-offs of IgA tTG antibody titers to reliably establish the diagnosis of CD in adults as well. To the best of our knowledge, no such study has been done in India so far. So, the aim of the present study was to assess whether IgA anti-tTG antibody levels above a certain limit could be considered sufficient for detecting duodenal mucosal abnormalities.

## Materials and methods

Study population

This study was done in the Department of Microbiology in collaboration with the Departments of Medical Gastroenterology and Pathology. We retrospectively analyzed the medical records of suspected CD patients who underwent small bowel biopsies over a period of 4.5 years (January 2019-July 2023). We excluded patients with age < 18 yrs, patients with Whipple’s disease, Crohn’s disease, and small bowel malignancy. Also, patients with no records of anti-tTG IgA antibodies were excluded from the study. So, a total of 94 patients’ records of biopsy confirmed CD adult patients with serology records available were included in the study group. The clinical profile and socio-demographic details of these patients were noted.

Laboratory methods

Serology

Serum samples from the study group were tested for IgA and IgG anti-tTG antibodies. The IgA and IgG anti-tTG antibodies were determined by quantitative indirect solid phase enzyme-linked immunosorbent assay (ELISA) kit which had a 100% specificity and 88.5% sensitivity with a confidence limit of 95%. The cut-off for the kit was 20 IU/mL. Positive and negative quality controls available in the kit were used while performing ELISA. The kit is based on human recombinant tTG antigen. A serological test for IgG Ab was done to rule out the possibility of IgA deficient false negative cases.

Histology

Multiple biopsies were taken from the second part of the duodenum through eosophagoduodenoscopy. Histopathological diagnosis was established on routine hematoxylin and eosin-stained sections and immunohistochemical (IHC) staining with antihuman CD3 antibody. The histopathological grading was performed as per modified Marsh grading [12]. Duodenal biopsy report with a level of Marsh 3 corresponding to complete villous atrophy was considered as the confirmatory diagnosis of CD [13].

Categorization of Patients

Two cut-offs for anti-tTG antibody titers were selected, on the basis of which patients were divided into two groups each.

First cut-off - 10 x ULN (200 IU/mL) - Group 1: ≥ 10 x ULN (> 200 IU/mL), Group 2: ≤ 10 x ULN (< 200 IU/mL).

Second cut-off - 5 x ULN (100 IU/mL) - Group 1: ≥ 5 x ULN (>100 IU/mL), Group 2: ≤ 5 x ULN (<100 IU/mL).

Statistical analysis

Sensitivity, specificity, NPV, and PPV for different cut-off values of tTG levels were calculated. Analysis of variance or Kruskal-Wallis test with appropriate post-hoc analysis was used to compare Marsh Grades with tTG titers. A p-value of <0.05 was considered statistically significant. The receiver operating curve (ROC) curve was constructed and the area under the curve (AUC) was calculated to determine the accuracy of tTG levels in predicting villous atrophy.

## Results

A total of 94 patient records fulfilled the criterion and were included in the study group. Among them, 58 were females and 36 were males. The median age of the study population was 33.5 yrs. A maximum number of patients (82%) presented with typical/gastrointestinal symptoms (including diarrhea as the most common symptom, abdominal pain, and vomiting). The rest of the patients presented with extraintestinal symptoms such as short stature, hypothyroidism, and thyroiditis. The preponderance of GI over atypical symptoms was statistically significant (p = 0.0001).

**Figure 1:**
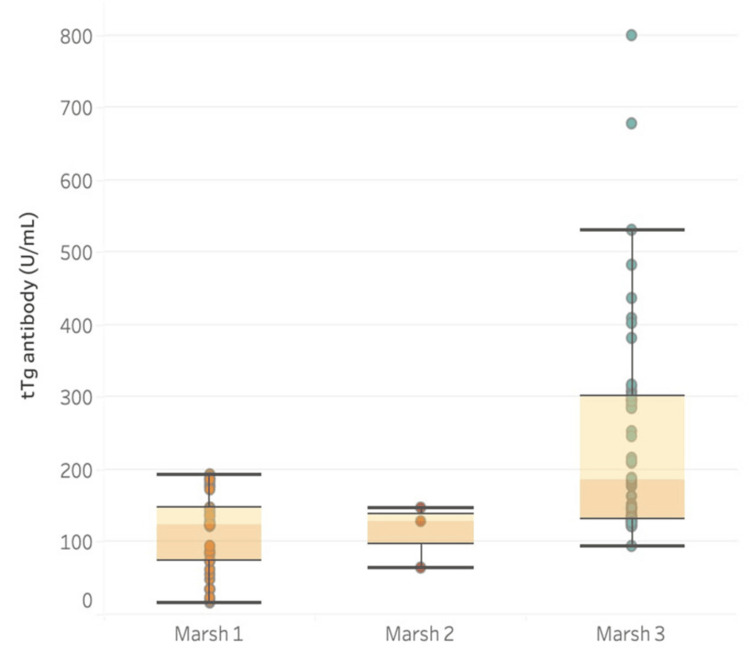
tTG titers against different Marsh grades in patients tTg Ab = Tissue transglutaminase antibody titers in IU/mL Histological Marsh grading 1, 2, 3 on small intestinal biopsy

**Figure 2:**
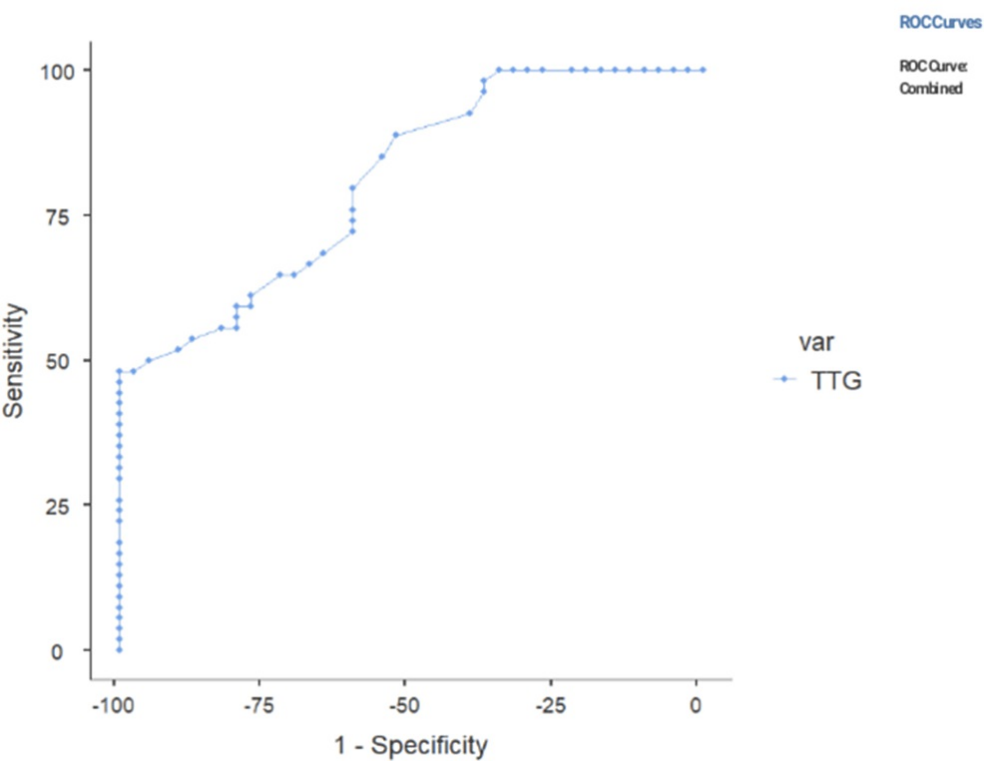
ROC curve analysis of tTG values against Marsh 3 histology ROC - Receiver operating characteristic curve x-axis - Specificity for detection of Marsh Grade 3 y-axis - Sensitivity for detection of Marsh Grade 3

Among 94 patients, 37 (39%) had Marsh Grade 1, three (3%) had Grade 2, and 54 (58%) had Grade 3. A number of patients with biopsy findings consistent with various Marsh Grading is shown in Figure 1. All patients with cut-off ≥200 had findings consistent with Marsh Grade 3. 72% of patients had anti-tTGA titers below 200 IU/mL, and 27.6% of patients had anti-tTGA titers above 200 IU/mL. Among 54 Marsh Grade 3 patients, 26 had a cut-off above 200 IU/mL (Table 1). The sensitivity, specificity, and positive predictive value (PPV) for detecting Marsh grade 3 lesions at cut-off 10 times the ULN is shown in Table 2. Another cut-off of anti-tTGA antibodies that were tested against various Marsh grades was 5 x ULN. At this cut-off, we had 53 patients in Grade 3, 23 patients in Grade 1, and 2 patients in Grade 2. The distribution of Marsh gradings according to ULN x 5 cut-off and performance characteristics for prediction are shown in Tables 3, 4, respectively. At this cut-off, the sensitivity increased by 1%, and specificity decreased from 100% to 37.50%. To predict the best possible cut-off that would retain the specificity with a reasonably good sensitivity, ROC curve analysis was done and is depicted in Figure 2. The cut-off that was achieved was 210 IU/mL.

**Table 1:** Distribution of no. of patients with various Marsh gradings as per ULN x 10 cut-off Anti-tTg Ab cut-off = Anti-tissue transglutaminase antibody levels IU = International unit/mL Marsh grading = Histological grading on small bowel biopsy

Anti tTG Ab Cut offs (Total no. of patients)	No. of patients with MARSH GRADE 3 (54)	No. of patients with MARSH GRADE 2 (3)	No. of patients with MARSH Grade 1 (37)
> 200 IU/ml (10XULN) (26)	26	0	0
< 200 IU/ml (10XULN) (68)	28	3	37

**Table 2:** Performance characteristics for prediction of intestinal lesions at ULN x 10 cut-off CI - Confidence Interval

Statistic	Value	95% CI
Sensitivity	48.15%	34.34% to 62.16%
Specificity	100.00%	29.24% to 100.00%
Positive Predictive Value	100.00%	86.77% to 100.00%
Negative Predictive Value	9.68%	7.65% to 12.17%

**Table 3:** Distribution of Marsh gradings acc to ULN x 5 cut-off Anti-tTg Ab cut-off = Anti-tissue transglutaminase antibody levels IU = International unit/mL Marsh grading = Histological grading on small bowel biopsy

Anti tTG Ab Cut offs	MARSH GRADE 3	MARSH GRADE 2	MARSH GRADE1
>100 IU/ml (68)	53	2	23
<100 IU/ml (16)	1	1	14

**Table 4:** Performance characteristics for prediction of Marsh grading at cut-off ULN x 5 CI - Confidence Interval

Statistic	Value	95% CI
Sensitivity	98.15%	90.11% to 99.95%
Specificity	37.50%	22.73% to 54.20%
Positive Predictive Value	67.95%	62.45% to 72.99%
Negative Predictive Value	93.75%	67.38% to 99.09%

## Discussion

The correct diagnosis for CD is very crucial because false positive results would imply an unnecessary GFD for an entire lifetime and false negative results will put untreated CD patients at risk of various potential complications including osteoporosis, infertility, cirrhosis, lymphoma, etc.

Biopsy has been an important tool in the diagnostic algorithm for CD, but in a country like India where even to date not more than 5% of CD patients are diagnosed and treated, probably because of limited resources and a lack of skilled professionals in primary care centers, we need to find more approachable, easily available, reliable and accurate diagnostic tests to mitigate the underdiagnosis of the disease.

Since 2012, four major guidelines have been published by various gastrointestinal organizations on CD diagnosis as depicted in Table 5 [6]. Among them, ESPHAGN and World Gastroenterological Association guidelines allow for diagnosis of the disease without the need for intestinal biopsy [6,7]. These guidelines suggest that if the anti-IgA tTG titers are >10 x ULN, it can be considered the acceptable criterion for diagnosis of CD in symptomatic patients. Since then, many researchers across the world have evaluated the same proposal in different settings. In our study, we tried to evaluate the levels of anti-IgA tTG above which the PPV of diagnosing Marsh Grade 3 CD in symptomatic patients would be 100%.

**Table 5:** Guidelines for diagnosing celiac disease by various gastrointestinal organizations

GUIDELINES	RECOMMENDATIONS
American College of Gastroenterology (ACG) 2013 [6]	Combination of both small intestinal biopsy and serologic tests
British Society of Gastroenterology [6]	For adult CD diagnosis suggest that serologic tests, either tTG, EMA, or DGP should be done as the first step in diagnosis, followed by small intestinal biopsy
European Society of Pediatric Gastroenterology, Hepatology and Nutrition (ESPGHAN)2012 [6]	Non-invasive method of diagnosing CD in paediatric patients.
World Gastroenterological Association [6]	Serologic tests including anti-tTG and/or anti-EMA, or anti-DGP for diagnosis and biopsy suggested but not considered mandatory for CD diagnosis

Similar to many other studies in the literature, we considered Marsh Grade 3 histology as our confirmatory diagnosis for CD because of the following.

1. It represents the hallmark pathological changes of CD [14-16].

2. According to the 1990 ESPGHAN criteria, the presence of Marsh 3 histology on a gluten-containing diet was considered the hallmark of CD [17].

3. Marsh 1 findings are not specific to CD and can be found in other diseases like H. pylori infection, Crohn’s disease, patients on NSAIDs, etc. [18].

4. The transition from Marsh 1 to Marsh 2 is rarely encountered [18].

This study included 94 symptomatic patients, out of which 26/94 (27.6%) patients had anti-tTG IgA levels above 200 IU/mL (>10ULN cut-off). All these 26 (100%) patients had Marsh Grade 3 lesions on histology. Among Grade 2 and Grade 1 lesions, there was no patient who had IgA anti-tTG antibodies cut-off above 10 x ULN. On statistical analysis, it was observed that specificity and positive predictive value for the detection of Marsh Grade 3 lesions by this cut-off was 100%. We had a total of 54 patients with Marsh Grade 3 lesions with 28 patients having IgA anti-tTG antibodies titers< 10 x ULN. So, although the specificity is high the sensitivity was only 48.15% for diagnosing CD at this cutoff. Our study is in concordance with various other studies, which support that a cut-off of >10 ULN of IgA anti-tTG antibodies can accurately diagnose Marsh Grade 3 lesions [14,19,20].

The disease prevalence within the study group is said to influence the PPV of a diagnostic test [21]. Penny et al. determined the predictive capacity of the 10×ULN threshold against the Marsh 3 lesions on duodenal biopsy in a different cohort of individuals including patients with high clinical suspension and low clinical suspension of CD and observed that the cut-off ≥10×ULN is equally good at predicting Marsh 3 lesions in the setting of both.14In our study, we tested this cut-off in a set of symptomatic individuals and the PPV for detecting Marsh Grade 3 lesions found to be 100%.

This high tTG threshold recommended by the ESPHAGN guidelines is to control for tTG assay variation. Many studies have suggested even lower cut-off values of IgA anti-tTG antibody levels for predicting the intestinal lesions in CD as shown in Table 6. Few of them observed that a lower cut-off would retain the diagnostic specificity with increased sensitivity [22-26].

**Table 6:** Studies citing lower cut-offs for predicting intestinal lesions ULN - Upper limit of normal PPV - Positive predictive value

STUDIES	CUT-OFFs	STATS
Holmes et al. [22]	8XULN	PPV-100%
Tortora et al. [23]	8XULN	PPV-100%
Zanini et al. [24]	5XULN	Specificity- 100%
Alessio et al. [25]	7XULN	Close to 100%
Di Tola et al. [26]	3.6XULN	PPV-97.2%

We also statistically analyzed another cut-off of 5ULN and observed that the sensitivity for detecting the Marsh 3 lesions increased significantly but the specificity dropped dramatically as Marsh 1 and 2 lesions were also included within this cut-off. So, ROC curve analysis was done to identify the best achievable cut-off for our kit which would retain the specificity with a reasonably good sensitivity. This cut-off came out to be 210 IU/mL, which retains the 100% specificity and has a sensitivity of 49.15% (sensitivity increased by only 1%).

Studies have demonstrated a wide discrepancy of the ULNs when comparing different commercially available anti-TG2 assays probably because of a lack of standardization between laboratories. So, the threshold ULN cut-off for different kits is specific for each of them and there needs to be a local validation by the laboratories.

Despite enough evidence supporting the hypothesis that tTG antibodies above a certain level may have a good PPV for diagnosing CD, adult gastroenterologists are still reluctant to put a patient on a GFD based on serological tests alone as there are no standard guidelines available. Even the British Society of Gastroenterology Guidelines for the diagnosis of CD still maintain that biopsy cannot be replaced by serology and is still essential for the diagnosis of CD [16]. There are a few studies in contrast to our study which also state that tTG levels cannot be relied on, and biopsies are mandatory for the diagnosis of CD [27].

Fleming et al state that the majority of patients who had positive serology for CD did not undergo an SBB, implying poor compliance with current guidelines which could be another reason for the under-diagnosis of the disease [11]. There is no data from India that shows the percentage of patients who undergo a biopsy following a positive serology test. Considering the other issues with biopsy such as variation in the interpretation of biopsies by histopathologists, presence of patchy lesions leading to misinterpretation of diagnosis, histological findings being characteristic but not specific for CD may increase the rate of false negative biopsy results. So, many more such studies need to be done to frame guidelines for the cut-off for IgA anti-tTGA antibodies above which the patient may be directly put on a GFD without further need for confirmation by biopsy.

Limitations of the study

ESPGHAN guidelines have included testing for endomysial antibody (EMA Ab) positivity in a second blood sample of the same patient to increase the predictive capacity of the “no biopsy” approach. However, at our center, there is no facility for immunofluorescence testing of EMA Ab as it is very costly and labor-intensive, so we were not able to include it in our testing algorithm.

## Conclusions

Biopsy-free approach may have a promising role in adult symptomatic patients with a higher cut-off for anti-tTg Ab especially in primary care settings with limitations for resources. Considering the fact that CD is a long-standing disease that can be controlled if rapid diagnosis and timely GFD is initiated, we must give consideration to a biopsy-free approach after meticulous interpretation of data from various parts of the country.
